# PDE5 Inhibitors as Potential Tools in the Treatment of Cystic Fibrosis

**DOI:** 10.3389/fphar.2012.00167

**Published:** 2012-09-18

**Authors:** Sabrina Noel, Barbara Dhooghe, Teresinha Leal

**Affiliations:** ^1^Louvain Centre for Toxicology and Applied Pharmacology, Institut de Recherche Expérimentale et Clinique, Secteur des Sciences de la Santé, Université Catholique de LouvainBrussels, Belgium

**Keywords:** CFTR, cystic fibrosis, PDE5 inhibitors, sildenafil, vardenafil, taladafil

## Abstract

Despite great advances in the understanding of the genetics and pathophysiology of cystic fibrosis (CF), there is still no cure for the disease. Using phosphodiesterase type 5 (PDE5) inhibitors, we and others have provided evidence of rescued F508del-CFTR trafficking and corrected deficient chloride transport activity. Studies using PDE5 inhibitors in mice homozygous for the clinically relevant F508del mutation have been conducted with the aim of restoring F508del-CFTR protein function. We demonstrated, by measuring transepithelial nasal potential difference in F508del mice following intraperitoneal injection of sildenafil, vardenafil, or taladafil at clinical doses are able to restore the decreased CFTR-dependent chloride transport across the nasal mucosa. Moreover, vardenafil, but not sildenafil, stimulates chloride transport through the normal CFTR protein. We developed a specific nebulizer setup for mice, with which we demonstrated, through a single inhalation of PDE5 inhibitors, local activation of CFTR protein in CF. Significant potential advantages of inhalation drug therapy over oral or intravenous routes include rapid onset of pharmacological action, reduced systemic secondary effects, and reduced effective drug doses compared to the drug delivered orally; this underlines the relevance and impact of our work for translational science. More recently, we analyzed the bronchoalveolar lavage of CF and wild-type mice for cell infiltrates and expression of pro-inflammatory cytokines and chemokines; we found that the CFTR activating effect of vardenafil, selected as a representative long-lasting PDE5 inhibitor, breaks the vicious circle of lung inflammation which plays a major role in morbi-mortality in CF. Our data highlight the potential use of PDE5 inhibitors in CF. Therapeutic approaches using clinically approved PDE5 inhibitors to address F508del-CFTR defects could speed up the development of new therapies for CF.

## Introduction

Approximately 80,000 people in the world are diagnosed with Cystic Fibrosis (CF), the most common, life-threatening, recessively inherited disease in Caucasian populations. Affecting about one newborn in every 2,500 live births, CF is due to mutations in the *CF transmembrane conductance regulator* (*CFTR*) gene (Kerem et al., 1989; Riordan et al., 1989) which encodes the main chloride channel expressed in epithelia. CF disease causes abnormal mucociliary clearance mainly in the lungs, leading to a vicious cycle of obstruction/infection/inflammation that progressively and irreversibly damages lung tissue and architecture. Many organs are affected in CF but pulmonary disease is the major cause of morbidity and mortality (Rowe et al., 2005; Davis, 2006). Although life expectancy and quality of life have progressively improved over time, there is still no cure for CF.

The most common disease allele, F508del, corresponding to a deletion of a single phenylalanine residue at position 508 of a single polypeptide chain of 1480 amino acids, prevents the efficient folding of the CFTR protein. The F508del-CFTR protein is correctly translated but it is retained in the endoplasmic reticulum and directed toward proteosomal degradation (Lukacs et al., 1994). As a consequence, expression of the misfolded, immature, partly glycosylated F508del-CFTR protein at apical membranes is reduced, leading to a loss-of-function of transepithelial chloride transport.

Recent research in CF basic science has focused on the discovery of pharmacological therapies directed to treat mutation-specific changes (for review, Lubamba et al., 2012a). In the case of the F508del-CFTR mutation, efforts have been made to correct localization of the mutant protein by favoring its expression at the apical membrane of cells. However, it has been recognized that rescuing F508del-CFTR to the plasma membrane does not completely correct chloride transport abnormalities as it also displays reduced channel activity (Amaral, 2004). Therefore, finding a compound that also promotes CFTR channel activity would be of a great benefit. Searching for such compounds, we and others have demonstrated the potential of inhibitors of phosphodiesterase type 5 (PDE5), such as sildenafil, vardenafil, and taladafil, for the treatment of CF. Indeed, recent findings have evidenced that the drugs, already in clinical use for the treatment of erectile dysfunction and of pulmonary arterial hypertension, are able to rescue F508del-CFTR trafficking (Dormer et al., 2005; Robert et al., 2008) and to improve its channel activity (Lubamba et al., 2008, 2011).

## Cyclic Nucleotide Phosphodiesterases

**Figure 1:**
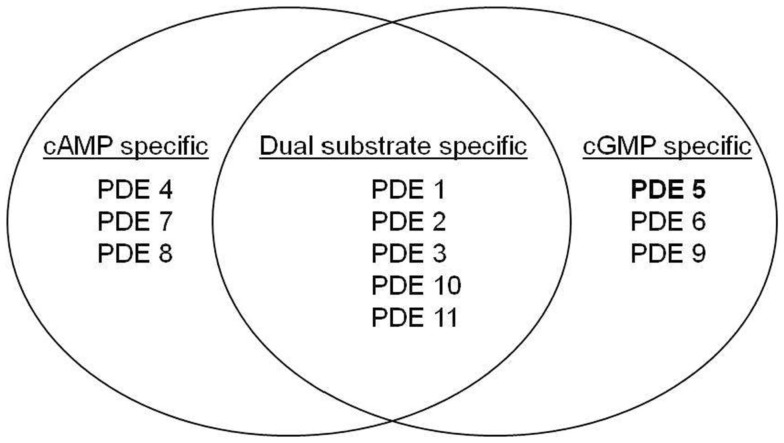
**Substrate specificity of the different families of PDE**. Although a high degree of homology has been observed within the catalytic domain of PDEs, slight structural differences in these domains determine the specificity of substrate of PDEs (Xu et al., 2004).

PDE activity is found in all cells, but with a distinct cellular and subcellular distribution of the 11 mammalian isoforms (Beavo et al., 1970). By catalyzing the hydrolysis of 3′ cyclic phosphate bonds of adenosine and/or guanosine 3′5′ cyclic monophosphate (cAMP and/or cGMP) the enzyme regulates the intracellular levels of the second messengers. The multiple isoforms of PDEs and their 50 or so subtypes, displaying different kinetics and regulatory properties (Cheung, 1970; Conti, 2000; Soderling and Beavo, 2000; Francis et al., 2001; Mehats et al., 2002), are characterized by their specificity and sensitivity to calcium-calmodulin and their affinity for cAMP or cGMP (Figure 1).

Eleven families of PDE have been identified in mammalian tissues (Cheung, 1970; Conti, 2000; Soderling and Beavo, 2000; Francis et al., 2001; Mehats et al., 2002) and are classified on the basis of their amino acid sequences, substrate specificities, pharmacological properties, and tissue distributions (Table 1).

**Table 1:** **Main characteristics of phosphodiesterase families, corresponding substrates and specific inhibitors, and their clinical applications**.

PDE	Main substrate	Km (μM) cAMP	Km (μM) GMP	Tissue expression	Specific inhibitors	Reference
1	Ca2 + / calmodulin-stimulated cAMP ≤ cGMP	70–120	0.6–6.0	Heart, brain, lung, smooth muscle, T lymphocytes, sperm	KS505a, bepril, Vinpocetine, Flunarizine, Amiodarone^a,b^	Bender and Beavo (2006), Yan et al. (1995), Loughney et al. (1996), Yu et al. (1997), Ahn et al. (1991), Medina et al. (2006), Menniti et al. (2006), Truss et al. (2001), Zhang et al. (2004), Jeon et al. (2010), Reed et al. (2002), Filgueiras et al. (2010), Medina (2010)
2	cAMP = cGMP	30	10–24	Adrenal gland, heart, lung, liver, platelets	EHNA, BAY 60–7550, Oxindole, PDP^c^	Rosman et al. (1997), Rivet-Bastide et al. (1997), Sadhu et al. (1999), Suvarna and O’Donnell (2002), Podzuweit et al. (1995), Repaske et al. (1992), Boess et al. (2004), Rutten et al. (2009)
3	cAMP > cGMP	0.2–0.4	0.02–0.2	Heart, lung, liver, kidney, oocytes, adipocytes, T lymphocytes, platelets, inflammatory cells	Cilostamide, Cilostazol, Enoxamone, Milrinone, Siguazodan^b,d^	Palmer and Maurice (2000), Vandecasteele et al. (2001), Shin et al. (2007), Nohria et al. (2003), Carev et al. (2010)
4	cAMP	1.5–10	–	Kidney, brain, liver, lung, smooth muscle, cardiovascular tissues, Sertoli cells inflammatory cells	Rolipram, Roflumilast, Cilomilast, Drotaverine, ibudilast^b,d,e^	Tenor et al. (1995a,b,c), Essayan (2001), Scott et al. (1991), O’Byrne and Gauvreau (2009)
5	cGMP	290	2.9–6.2	Lung, platelets, vascular, smooth muscle	Sildenafil, Vardenafil, Tadalafil, Zaprinast^b,d,e^	Hamet and Coquil (1978), Coquil et al. (1980), Francis et al. (1980), Francis and Corbin (1988), Moncada and Martin (1993), Sebkhi et al. (2003), Ghofrani et al. (2006), Milligan et al. (2002), Nichols et al. (2002), Muirhead et al. (2002), Burgess et al. (2008), Klotz et al. (2001), Gresser and Gleiter (2002), Stark et al. (2001), Ormrod et al. (2002), Eardley and Cartledge (2002), Bella and Brock (2003), Staab et al. (2004), Brock (2003), Porst et al. (2003), Curran and Keating (2003), Corbin et al. (2005), Wharton et al. (2005), Prickaerts et al. (2002), Baratti and Boccia (1999)
6	cGMP	610–700	15–17	Photoreceptor	Dipyridamole	Zhang et al. (2005), Estrade et al. (1998)
7	cAMP	0.03–0.2	–	Skeletal muscle, heart, kidney, brain, pancreas, T lymphocytes, eosinophils, neutrophils	BRL-50481, BC30^b^	Gardner et al. (2000), Sasaki et al. (2000), Hetman et al. (2000a), Smith et al. (2003), Pitts et al. (2004), Vergne et al. (2004), Zhang et al. (2008)
8	cAMP	0.06	–	Testis, eye, liver, skeletal muscle, heart, kidney, ovary, brain, T lymphocytes	PF-04957325^f^	Perez-Torres et al. (2003), Wang et al. (2001), Hayashi et al. (2007), Kobayashi et al. (2003), Glavas et al. (2001), Dong et al. (2006), Vasta et al. (2006), Vang et al. (2010), Tsai et al. (2011), Dov et al. (2008)
9	cGMP	230	0.2–0.7	Kidney, liver, lung, brain, spleen, small intestine	BAY 73-6691	Soderling et al. (1998a,b), van der Staay et al. (2008)
10	cAMP < cGMP	0.2–1.0	13–14	Testis, brain	pyrazoloquinoline analogs	Soderling et al. (1999), Fujishige et al. (1999), Loughney et al. (1999), Hebb et al. (2004), Yang et al. (2012)
11	cAMP = cGMP	2.0–3.2	0.95–2.1	Skeletal muscle, prostate, kidney, liver, pituitary, testis, salivary glands	BC 11-38	Fawcett et al. (2000), Hetman et al. (2000b), Weeks et al. (2007), Ceyhan et al. (2012)

*^a^Therapeutic action with neuronal effects (neural plasticity, memory loss, detrusor instabilities, and urgency incontinence,…); ^b^Therapeutic action with anti-inflammatory effects; ^c^Without therapeutic action; ^d^therapeutic action for lung diseases (asthma, COPD); ^e^Therapeutic action with cardiovascular effects (inotopic, vasodilator,…); ^f^Therapeutic action for adrenal insufficiency*.

## PDE Inhibitors: Main Characteristics and Clinical Applications

Inhibition of PDEs leads to increasing intracellular concentrations of endogenous cAMP/cGMP (Bender and Beavo, 2006). Therefore, inhibition of PDE can mediate a variety of physiological mechanisms at different cell and organ levels. Strategies directed to promote inhibition of PDE activity have been applied as therapeutic tools in a variety of lung and inflammatory disorders, such as asthma and chronic obstructive pulmonary disease (COPD) but also in neuronal, cardiovascular, and other conditions (Table 1).

Many selective and non-selective PDE inhibitors have been explored as therapeutic agents. PDE1s are calcium- and calmodulin-dependent activators or regulators (Ahn et al., 1991; Yan et al., 1995; Loughney et al., 1996; Yu et al., 1997). Several isoforms have been recognized exhibiting different affinities for cAMP and cGMP. PDE1 inhibition has been investigated in treating neuronal plasticity (Medina et al., 2006; Menniti et al., 2006), detrusor instablities and urgency incontinence (Truss et al., 2001), memory loss (Zhang et al., 2004), reversal of the effects of early alcohol exposure in learning performance in the water maze (Jeon et al., 2010) and Parkinson and Alzheimer diseases (Reed et al., 2002). It was recently demonstrated that vinpocetine has a strong anti-inflammatory effect (Filgueiras et al., 2010; Medina, 2010).

PDE2, which metabolizes both cGMP and cAMP (Rosman et al., 1997), is highly expressed in heart (Rivet-Bastide et al., 1997) and brain but lower expression levels are found in a variety of organs (Sadhu et al., 1999). PDE2 inhibitors identified so far lack therapeutic actions (Repaske et al., 1992; Podzuweit et al., 1995; Suvarna and O’Donnell, 2002; Boess et al., 2004; Rutten et al., 2009).

It is well known that methylxanthines, non-selective PDEs found in tea, coffee, and cocoa, stimulate the central nervous system, relax the bronchial smooth muscle, and stimulate cardiac muscle. Methylxanthines have long been used as therapeutic agents in respiratory diseases (Sullivan et al., 1994; Barnes, 2003a,b,c; Bhatt-Mehta and Schumacher, 2003; Barnes and Stockley, 2005; Muller and Jacobson, 2011). Indeed theophylline (1,3-dimethylxanthine) and other methylxanthines have been used in medical practice long before they were identified as PDE inhibitors. Caffeine has long been used as a bronchodilating agent. It has been perceived that theophylline has additional anti-inflammatory properties for use in asthma or COPD, diseases characterized by inflammatory and immune responses. Paraxanthine (1,7-dimethylxanthine), the primary metabolite of caffeine (1,3,7-trimethylxanthine), acts through the ryanodine receptor to elevate intracellular calcium concentration and increases viability of neuronal cells in culture (Guerreiro et al., 2008). The synthesized 3-isobutyl-1-methylxanthine (IBMX) has a much higher affinity for PDEs and, at low concentrations, it preferentially inhibits cGMP-dependent over cAMP-dependent PDEs (Wells et al., 1975). Moreover, methyxanthines are potent antagonists of adenosine receptors (Muller and Jacobson, 2011).

PDE3 are non-selective enzymes with high affinity for both cAMP and cGMP (Palmer and Maurice, 2000). A large number of selective PDE3 inhibitors including milrinone, cilostamide, and cilostazol have been identified as potential therapeutic tools for cardiovascular diseases and asthma (Vandecasteele et al., 2001; Nohria et al., 2003; Shin et al., 2007; Carev et al., 2010).

PDE4s have high affinity for cAMP, they are expressed in inflammatory cells such as T and B lymphocytes, eosinophils, neutrophils, airway epithelial cells and endothelial cells (Tenor et al., 1995a,b,c), cardiovascular tissues, and smooth muscles. PDE4 inhibitors have been developed for the treatment of asthma and COPD (Essayan, 2001). Rolipram, a highly selective first generation PDE4 inhibitor, has been used for many years as a research tool to investigate the role of PDE4. Rolipram inhibits neutrophilic and eosinophilic inflammation; it proved to be an effective antidepressant, but side effects such as nausea and gastro-intestinal disturbance terminated its clinical development (Scott et al., 1991). Roflumilast was beneficial, as assessed by improvement in lung function, even when added to a long acting β_2_ agonist or a long acting inhaled antimuscarinic (O’Byrne and Gauvreau, 2009).

**Figure 2:**
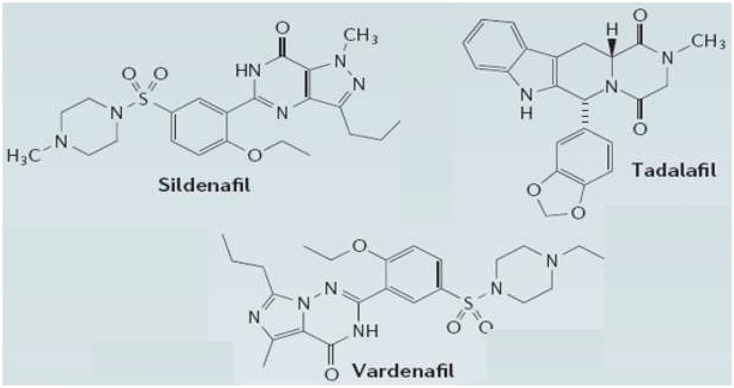
**Structures of the three clinically approved phosphodiesterase type 5 inhibitors**. Sildenafil, vardenafil, and tadalafil have been approved for treatment of erectile dysfunction. Sildenafil and taladafil have also been approved as a treatment for pulmonary arterial hypertension. Sildenafil citrate is designated chemically as 1-[[3-(6,7-dihydro-1-methyl-7-oxo-3-propyl-1*H*pyrazolo[4,3-*d*]pyrimidin-5-yl)-4-ethoxyphenyl]sulfonyl]-4-methylpiperazine citrate. Vardenafil HCl is designated chemically as piperazine, 1-[[3-(1,4-dihydro-5-methyl-4-oxo-7-propylimidazo[5,1-*f*][1,2,4]triazin-2-yl)-4-ethoxyphenyl]sulfonyl]-4-ethyl-, monohydrochloride. Taladafil is designated chemically as pyrazino[1′,2′:1,6]pyrido[3,4-b]indole-1,4-dione,6-(1,3-benzodioxol-5-yl)-2,3,6,7,12,12a-hexahydro-2-methyl-, (6R,12aR)-.

PDE5 has a higher affinity for cGMP and was identified in rat platelets (Hamet and Coquil, 1978; Coquil et al., 1980) and rat lung (Francis et al., 1980; Francis and Corbin, 1988). It is known to be abundant in smooth muscle cells (Moncada and Martin, 1993) and high expression levels have been found in pulmonary vascular smooth muscle, bronchial blood vessels, and airway smooth muscle (Francis et al., 1980; Francis and Corbin, 1988). Recent data have shown that PDE5 may modulate pressure-induced cardiac hypertrophy and fibrosis (Sebkhi et al., 2003). Several compounds that potently inhibit PDE5 have been synthesized recently, and three of these are currently in clinical use for male erectile dysfunction (Figure 2). Sildenafil (Viagra; Pfizer Inc., USA), the first compound of this class to be marketed, provides well-tolerated pharmacotherapy for erectile dysfunction (Milligan et al., 2002; Muirhead et al., 2002; Nichols et al., 2002; Ghofrani et al., 2006; Burgess et al., 2008). Two newer selective PDE5 inhibitors, vardenafil (Levitra; GlaxoSmithKline, UK; Klotz et al., 2001; Stark et al., 2001; Gresser and Gleiter, 2002; Ormrod et al., 2002), and taladafil (Cialis; Eli Lilly, US; Eardley and Cartledge, 2002; Bella and Brock, 2003; Brock, 2003; Curran and Keating, 2003; Porst et al., 2003; Staab et al., 2004) have the same mechanism of action, as they specifically bind to the catalytic site of the enzyme catalyzing the breakdown to 5′-GMP of cGMP, the second messenger of the nitric oxide (NO) pathway in vascular smooth muscle cells (Moncada and Martin, 1993). Sildenafil (under the tradename Revatio) and taladafil (under the tradename Adcirca) have also been approved for the treatment of ailments related to smooth muscle tissues, such as pulmonary arterial hypertension (Sebkhi et al., 2003; Corbin et al., 2005; Wharton et al., 2005). It has been reported that sildenafil and vardenafil raise hippocampal cGMP levels and improve memory in aged rats (Prickaerts et al., 2002) and mice (Baratti and Boccia, 1999).

PDE6s display high affinity for cGMP and are expressed in the photoreceptor outer segments of the mammalian retina, in which they mediate transduction of the light signal into an electrical signal (Zhang et al., 2005). Dipyridamole has been described to be a very potent cGMP-specific PDE inhibitor of visual transduction by cGMP accumulation (Estrade et al., 1998).

PDE7s are characterized by their high affinity and selectivity for cAMP as a substrate (Gardner et al., 2000; Hetman et al., 2000a; Sasaki et al., 2000; Smith et al., 2003; Pitts et al., 2004; Vergne et al., 2004; Zhang et al., 2008). Expression is abundant in T cells, eosinophils and neutrophils, epithelial cells, vascular smooth muscle cells, and lung fibroblasts (Smith et al., 2003). Several distinct PDE7 inhibitors have been reported (Pitts et al., 2004; Vergne et al., 2004). As PDE7 is simultaneously expressed in inflammatory cells and in the brain highlights the potential role of PDE7 as drug target for neuroinflammation. It has been shown that selective PDE7 inhibition or dual PDE4/7 inhibition may provide a novel therapeutic approach for the treatment of chronic lymphocytic leukemia (CLL) by enhancing killing and increasing specificity for CLL cells (Zhang et al., 2008).

PDE8s are cAMP specific, widely distributed in various tissues (Glavas et al., 2001; Wang et al., 2001; Kobayashi et al., 2003; Perez-Torres et al., 2003; Dong et al., 2006; Hayashi et al., 2007) and abundant in testis (Vasta et al., 2006). The company Pfizer reported on a small molecule called PF-04957325 that selectively inhibits PDE8 at very low doses (Vang et al., 2010). PDE8-selective inhibitors might be used to correct adrenal insufficiency, and a PDE8 activator might be used to treat Cushing’s syndrome (Tsai et al., 2011). It has also been shown that inhibiting PDE8 potentiates the biphasic insulin response to glucose (Dov et al., 2008).

PDE9 is one of the most recently discovered PDE families. It has a very high affinity for cGMP and it is expressed in a variety of tissues (Soderling et al., 1998a,b). Compared to the other cGMP-specific PDEs, PDE9 apparently lacks the non-catalytic cGMP-binding domain present in the cGMP-specific PDE5 and PDE6 and also in the dually specific PDE2. BAY 73-6691, acting as a PDE inhibitor selective for the PDE9A subtype, is a drug developed by Bayer for the treatment of Alzheimer’s disease (van der Staay et al., 2008).

PDE10 was isolated and characterized as a dual-substrate gene family distributed in fetal lungs and brain (Fujishige et al., 1999; Loughney et al., 1999; Soderling et al., 1999). The finding that striatal PDE10 mRNA and protein levels have been found to be reduced in Huntington’s disease (Hebb et al., 2004) would impact on the development of PDE10 agonists. Based on their high expression levels in the brain, PDE10s have become a target for central nervous system research, especially concerning cognitive deficits related to schizophrenia and psychotic statuses. A series of pyrazoloquinoline analogs have been synthesized and shown to bind with high affinity to PDE10 (Yang et al., 2012).

PDE11 are characterized by their high affinity for both cAMP and cGMP, although kinetic characteristics for the variants are different (Fawcett et al., 2000; Hetman et al., 2000b; Weeks et al., 2007). BC 11–38 is a recently identified potent and selective PDE11 inhibitor (IC_50_ = 0.28 μM) with potential application for adrenal insufficiency (Ceyhan et al., 2012).

## PDE Inhibitors as Potential Tools in the Treatment of Cystic Fibrosis

As an important second messenger signaling molecule, cAMP controls a wide variety of eukaryotic and prokaryotic responses to extracellular cues (Antoni, 2000). As CF is characterized by a defective cAMP-dependent chloride conductance in epithelial cells, it could be expected that modulating intracellular levels of the second messenger would bring beneficial therapeutic effects for patients with CF.

### Non-selective PDE inhibitors

Non-specific PDE inhibitors such as IBMX, theophylline, and DPMX (7-methyl-1,3-dipropylxanthine) have been shown to activate normal and mutated CFTR chloride channels in epithelia (Chappe et al., 1998). Due to impact on the cAMP pathway and activity at low concentrations, studies have looked at the effect of methylxanthines on the cAMP activated CFTR channel. It has been reported that IBMX increases CFTR chloride current in *Xenopus* oocytes expressing F508del-CFTR (Drumm et al., 1991). In nasal bronchial epithelial tissues expressing the mutant F508del-CFTR, treatment with IBMX associated with a potent adenylate cyclase agonist, forskolin was unable to stimulate chloride efflux (Grubb et al., 1993). However, stably transfected F508del-CFTR cells (Haws et al., 1996) showed a sevenfold increase in cAMP levels following IBMX treatment but not after cyclopentyl-1,3-dipropylxanthine (CPX), another non-specific PDE inhibitor. Interestingly both IBMX and CPX potentiated the effect of forskolin on CFTR-mediated efflux of ^125^I by 2.5-fold (Haws et al., 1996). A potentiation by IBMX of prostaglandin E (PGE)-induced bicarbonate secretion has been reported in the rat duodenum *in vivo* (Takeuchi et al., 1997; Aoi et al., 2004).

### Selective PDE inhibitors

PDE inhibitors increase cAMP by inhibiting one or more enzymes involved in cAMP degradation. Cyclic AMP-activated PKA mediates phosphorylation of CFTR and increases the open probability of the CFTR channel. PDE3 inhibitors, amrinone, and milrinone, also cause vasodilation, which may be beneficial for CF airways. Drumm et al. showed that inhibiting PDE had a larger effect on CFTR activation than have adenylate cyclase stimulants (Kelley et al., 1995). Using airway epithelial cell lines expressing wild-type CFTR, Calu-3, and 16HBE cells, it has been found that, at 100 μM concentrations, milrinone, or amrinone applied in the absence of adenylate cyclase activators, stimulate chloride efflux by 13.7-fold (Kelley et al., 1995). No effect on chloride efflux was found under stimulation with IBMX, rolipram, or dipyridamole. The increase of channel efflux by PDE3 inhibitor, amrinone, or milrinone, was not associated with a significant rise in cAMP concentrations but it was inhibited by protein kinase A inhibitors (H-8 and Rp-cAMPS), suggesting that it might work through a more distal signal. Kelley et al. (1996) also looked at endogenous CFTR in transformed nasal polyp tissue of patients homozygous for F508del (CF-T43). They found that, when administered in the presence of a β-agonist (isoproterenol) and protein kinase A activator, milrinone, and amrinone, at 100 μM concentrations, increased chloride efflux by 19–61% from baseline. Mice homozygous for F508del-CFTR were administered with a combination of milrinone (100 μM) and forskolin (10 μM; Kelley et al., 1997). This combination of drugs resulted in an increased magnitude of the nasal potential difference. The implications of this study are exciting; but the effect was confirmed in mice but not in humans (Smith et al., 1999).

It has been shown that CFTR has a major role in the regulation of duodenal bicarbonate secretion (Hogan et al., 1997). Furthermore, O’Grady et al. (2002) showed that both PDE1 and PDE3 are involved in the activation of CFTR in T84 cells and human colonic epithelial cells. Hayashi et al. (2007) suggested that PDE1 and PDE3 are involved in the regulation of duodenal bicarbonate secretion and that the response to PGE2 is associated with both PDE1 and PDE3, while the response to NO is mainly modulated by PDE1 (Hayashi et al., 2007). McPherson et al. (1999) showed that a selective cyclic nucleotide PDE5 inhibitor partially corrected defective l-adrenergic stimulation of mucin secretion in CFTR antibody-inhibited submandibular cells. The PDE5 inhibitor did not increase cAMP levels, nor did it potentiate isoproterenol-induced cAMP rise (McPherson et al., 1999). Of note, Dormer et al. (2005) demonstrated that the PDE5 inhibitor sildenafil also acts as a pharmacological chaperone. Because sildenafil is approved for clinical use, they speculated that their data might speed up the development of new therapies for CF (Dormer et al., 2005).

## Comparison of the PDE5 Inhibitors

There are distinct differences between the three clinically approved PDE5 inhibitors, sildenafil, vardenafil, and tadalafil, regarding their selectivity and specificity for PDE inhibition, with consequences on safety profile but also on biopharmaceutic and pharmacokinetic disparities that largely affect efficacy of the compounds (Klotz et al., 2001; Gresser and Gleiter, 2002; Milligan et al., 2002; Muirhead et al., 2002; Nichols et al., 2002; Burgess et al., 2008). Sildenafil and vardenafil are very similar in terms of chemical structure, whereas tadalafil, with a methyldione structure, differs markedly (Figure 2). These chemical properties are also reflected in similarities and dissimilarities of their clinical pharmacokinetics.

PDE5 inhibitors are rapidly absorbed after oral administration, with peak concentrations reached slightly earlier for vardenafil compared to sildenafil and tadalafil (Klotz et al., 2001; Gresser and Gleiter, 2002; Milligan et al., 2002; Muirhead et al., 2002; Nichols et al., 2002; Burgess et al., 2008). Although no clear concentration-effect relationships have been established for any of the three PDE5 inhibitors, rapid absorption is considered essential for a rapid onset of efficacy. Administration of a high-fat meal had no significant effect on the rate and extent of absorption of tadalafil but decreased the rate of absorption for sildenafil and vardenafil. All three drugs are lipophilic and have a volume of distribution larger than the volume of total body water, indicating tissue uptake and binding. Furthermore, the three compounds are highly protein bound, with free plasma concentration fractions of only 4–6%.

The major route of elimination for all PDE5 inhibitors is hepatic metabolism, with renal excretion of unchanged drug accounting for 1% or less of the elimination pathways. Based on their relatively high systemic clearance after intravenous administration, sildenafil, and vardenafil can be classified as non-restrictively cleared drugs with intermediate to high hepatic extraction ratio. The relatively comparable distribution volumes together with the substantial differences in systemic clearance among the PDE5 inhibitors result in distinct differences of the elimination half-life, 3–5 h for sildenafil and vardenafil compared to 17.5 h for tadalafil. Tadalafil, however, has been detected in plasma even 5 days after oral administration, in line with its long half-life. This suggests the possibility of accumulation if taken regularly and in short intervals, which may result in an increased risk of side effects with excessive use.

## PDE5 Inhibitors for the Treatment of Cystic Fibrosis

So far, many efforts have been focused on CFTR pharmacotherapy to target the abnormal protein pharmacologically by various approaches such as direct correction of stop codon mutations, CFTR channel activation, or trafficking defects. High-throughput screening has been used to identify molecules that increase F508del-CFTR activity (Pedemonte et al., 2005; Van Goor et al., 2006; Carlile et al., 2007). Such molecules have been categorized according to whether they improve the folding/cellular processing defect (correctors) or increase the responsiveness of F508del-CFTR channels already present in the membrane to cAMP activation (potentiators). Sildenafil has been initially shown to correct F508del-CFTR processing when used at supratherapeutic doses (Dormer et al., 2005).

### PDE5 inibitors correct transepithelial chloride transport in cystic fibrosis: Parenteral administration

To test the hypothesis that PDE5 inhibitors sildenafil, vardenafil, and taladafil, when applied at therapeutic doses, are able to restore transepithelial ion transport abnormalities of the F508del-CFTR protein, we have conducted experimental studies (Lubamba et al., 2008, 2011) in CF mice homozygous for the F508del mutation (van Doorninck et al., 1995) and in their corresponding wild-type homozygous normal mice. The F508del-CFTR mouse model has been chosen because F508del is the most common and one of the most severe CF mutations and because the mouse model recapitulates, although with different degrees of severity in the different systems, the human disease phenotype. Epithelia of the F508del-CF mouse model are characterized by defective electrolyte transport, and *Pseudomonas aeruginosa* lipopolysaccharide (LPS) exposure mimics several aspects of CF airway epithelial inflammation such as increased pro-inflammatory cytokines, most notably interleukin (IL)-8, IL-6, and Tumor Necrosis Factor (TNF)-α, and the predominant neutrophil infiltration.

**Figure 3:**
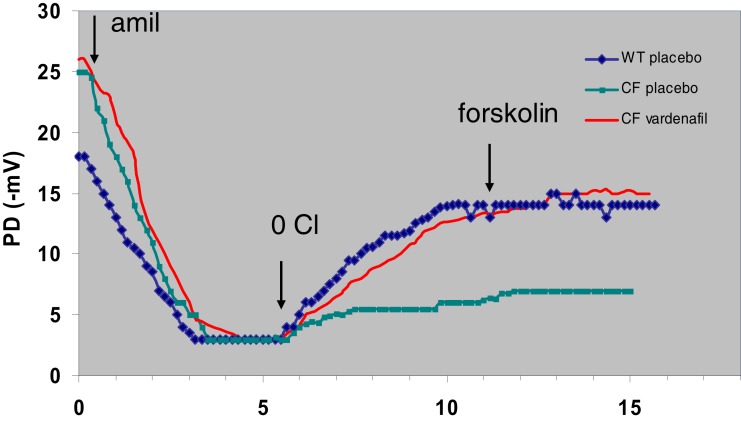
**Representative tracings of nasal potential difference (PD) measurements in wild-type (WT) and F508del-CF (CF) mice 24 h after placebo (saline) or vardenafil (single i.p. dose of 0.14 mg/kg body weight)**. Tracings show sequential response of the nasal surface to perfusion successively with basal solution, basal solution with 10^−4^ M amiloride (amil), chloride-free solution plus amiloride (0 Cl), and chloride-free solution with amiloride plus 10–5 M forskolin (forskolin). Arrows indicate change of solutions. As illustrated, basal values and amiloride response are not influenced by vardenafil treatment. However, chloride secretion (difference between values obtained at the end of the test and the end of the amiloride phase) is restored in CF animals and the effect lasts at least 24 h after vardenafil treatment (Lubamba et al., 2008).

**Figure 4:**
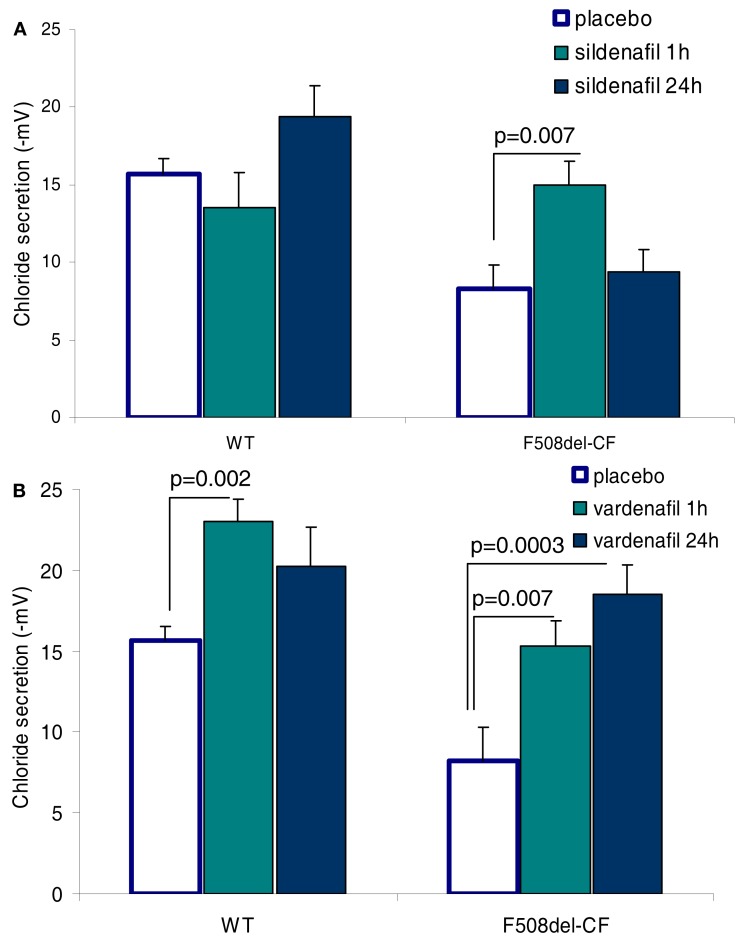
**Effect of parenteral (i.p.) administration of sildenafil (A; 0.7 mg/kg body weight) and of vardenafil (B; 0.14 mg/kg body weight) on CFTR-dependent chloride secretion assessed by means of the nasal potential difference (PD) in wild-type (WT) and F508del-CF mice**. Vardenafil stimulates chloride secretion of the wild-type CFTR. The correcting effect of vardenafil lasts at least 24 h. Data are expressed as mean ± SEM of 14–15 placebo treated animals and six animals treated with PDE5 inhibitors (Lubamba et al., 2008).

In our protocols, CFTR function has been assessed *in vivo* by measuring the transepithelial nasal potential difference, a diagnostic technique that has been more recently used as an index of therapeutic efficacy in novel fundamental therapies, either in animal models (Lubamba et al., 2008, 2009, 2011) or in CF patients (Sermet-Gaudelus et al., 2010; Leonard et al., 2012a,b). Our results provide clear evidence that intraperitoneal injection of PDE5 inhibitors (Figure 3), at therapeutic doses, to F508del-CF mice interact with CFTR, propping open the mutant protein to allow a normal flow of chloride ions across the epithelium of nasal mucosa, thereby restoring the decreased or even abolished CFTR-dependent chloride transport (Lubamba et al., 2008). In F508del mice, but not in *cftr* knockout mice, the chloride conductance, evaluated by perfusing the nasal mucosa with a chloride-free solution in the presence of amiloride (to inhibit sodium entry through the epithelial sodium channel ENaC) and with forskolin, is corrected 1 h after a single sildenafil administration (Figure 4A). A more prolonged effect, persisting for at least 24 h, is observed with vardenafil (Figure 4B). Moreover, vardenafil, but not sildenafil, is able to stimulate chloride transport associated with normal wild-type CFTR protein (Figure 4B). The forskolin response is increased after treatment with sildenafil or vardenafil in wild-type and in F508del mutant animals. In F508del mice, the chloride conductance in the presence of 200 μM DIDS (4-4′-diisothiocyanostilbene-2,2′-disulphonic acid), an inhibitor of alternative chloride channels, was much higher after sildenafil injection than following placebo treatment. This observation, in addition to the finding that no activating effect of chloride transport can be observed after treatment with PDE5 inhibitors in animals knockout for the CFTR protein, indicates that the action of PDE5 inhibitors on chloride transport across the respiratory epithelium involves F508del-CFTR and not a CFTR bypass channel. No effect on the sodium conductance was detected in any group of animals.

### PDE5 inhibitors correct transepithelial chloride transport in cystic fibrosis: Inhalational administration

More recently, animal studies have shown that nebulizing F508del-CF mice with any of the PDE5 inhibitors sildenafil, vardenafil, or taladafil led to correction of the nasal chloride transport (Lubamba et al., 2011). Correction is largest with taladafil and smallest, but still highly significant, with sildenafil. The effect of vardenafil, but not sildenafil, lasts at least 8 h after a single inhaled therapeutic dose. These findings clearly identify the inhalational route as a potential therapy for PDE5 inhibitors in CF which is clinically relevant taking into account the cost of systemic side effects of the drugs (Dalby and Suman, 2003).

Consistent with our results, it has recently been demonstrated that the inhalation route of administration for vardenafil is associated with an acceptable safety profile. Apart from brief coughing on inspiration, no clinically significant changes in blood pressure or heart rate and no serious adverse events were recorded (Berry et al., 2009). Inhalation drug therapy has several potential advantages over oral and intravenous routes, including rapid onset of pharmacological action, minimized systemic adverse effects and reduced effective drug doses compared to the same drug delivered orally (Berry et al., 2009); this greatly highlights the impact of our work for translational science.

### PDE5 inhibitors attenuate exaggerated inflammatory responses in cystic fibrosis

Another important goal of mutation-specific CF treatment is attenuation of exaggerated lung inflammatory responses (Legssyer et al., 2006; Gavilanes et al., 2009; Meyer et al., 2009). As lung inflammation plays a major role in morbi-mortality in CF, identifying a therapeutic strategy that combines ability to correct the basic ion transport defect and to reduce dysregulated inflammatory responses is very exciting and promising. It has been reported that sildenafil reduces neutrophil lung infiltration in murine airways infected with *P. aeruginosa* (Poschet et al., 2007). In addition, toxicological studies have shown that sildenafil pretreatment attenuates acrolein-triggered airway inflammation associated with mucin overproduction (Wang et al., 2009).

**Figure 5:**
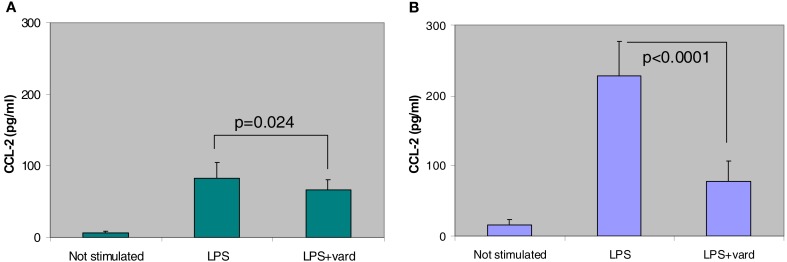
**Anti-inflammatory effect of *in vivo* treatment, by i.p. injection, of a single therapeutic dose of vardenafil (vard) to wild-type (A) and F508del-CF (B) mice on the inflammatory response induced by lipopolyssaccharide from *P. aeruginosa* (LPS)**. Biosynthesis of CCL-2 is significantly reduced in the bronchoalveolar lavage of vardenafil-treated CF and non-CF animals (Lubamba et al., 2012b).

More recently, we have found that vardenafil, selected as a representative PDE5 inhibitor for its longer-lasting CFTR activating effect, modulates the vicious circle of lung inflammation and attenuates the expression of pro-inflammatory cytokines and chemokines and cell infiltrates in the bronchoalveolar lavage (BAL) of CF and wild-type mice (Lubamba et al., 2012b). Intraperitoneal administration of a single pharmacological dose (0.14 mg/kg body weight) of vardenafil is followed by a reducing response in cell infiltrate and in the biosynthesis of several biomarkers of the inflammatory response. Most notably, levels of CCL-2 (chemokine C-C motif ligand), a cytokine playing a key role in the contribution of macrophages in the inflammatory response (Meyer et al., 2009), are significantly reduced in the BAL fluid after vardenafil treatment, particularly in CF animals (Figure 5).

The mechanism of action of vardenafil as an anti-inflammatory agent in CF as well as the target-effector cells involved in these responses are under investigation by our group. Altogether, our data indicate that PDE5 inhibitors have a strong therapeutic potential for treating CF. A clinical trial aimed at investigating the safety and efficacy of sildenafil in CF lung disease is currently listed on www.clinicaltrials.gov (NCT00659529).

### Perspective future research

Beside the clinical application for erectile dysfunction and for pulmonary arterial hypertension, a growing body of research has confirmed putative beneficial effects of PDE5 inhibitors in CF. Recent studies conducted in F508del and in wild-type CFTR expressing *Xenopus laevis* oocytes and human bronchial epithelial cells have indicated that sildenafil acts as a corrector and as a potentiator of the mutant and wild-type protein by distinct cGMP-independent and cGMP-dependent mechanisms respectively (Leier et al., 2012). While in *X. laevis* oocytes, low (1.5 μmol/l) doses were required to rescue F508del-CFTR function and cell membrane localization, suprapharmacological doses roughly 120 times larger than those commonly used for the treatment of erectile dysfunction were needed to achieve the same correcting effects in human bronchial epithelial cells (Leier et al., 2012). In this perspective, adverse drug effects including flushing, headache, and other cardiovascular effects could compromise the potential use of PDE5 inhibitors in CF.

Attempts should therefore be made either to achieve chemical modifications of PDE5 inhibitors with enhanced biochemical potency and selectivity or to allow inhalational therapy of the drugs. A structural analog of sildenafil, KM11060, designated chemically as 7-chloro-4-{4-[4-chlorophenyl)sulfonyl]piperazino}quinoline, has been recently identified as a novel potent corrector of the F508del-CFTR trafficking defect (Robert et al., 2008). F508del-CFTR trafficking was partially restored and maturation of the mutant protein was significantly increased in baby hamster kidney cells treated with low doses for a short duration (10 nM for 24 h or 10 μM for 2 h) of the compound (Robert et al., 2008). Since the morbi-mortality of CF is mostly related with respiratory manifestations and an acceptable safety profile with no serious adverse events was recorded when vardenafil was applied by inhalational route (Berry et al., 2009), topical airway deposition of PDE5 inhibitors (Lubamba et al., 2011) should be considered in future human studies. As a matter of fact, inhalation drug therapy has several potential advantages over oral and intravenous routes, including rapid onset of pharmacological action, minimized systemic adverse effects, and reduced effective drug doses compared to the same drug delivered orally (Dalby and Suman, 2003).

## Conclusion

Despite great advances in the understanding of the genetics and pathophysiology of the disease, there is still no cure for CF and existing therapies have mainly aimed at alleviating clinical symptoms. Recent experimental evidence has highlighted the potential of PDE5 inhibitors, sildenafil, vardenafil, and taladafil, as therapeutic agents in CF. As the drugs are able to correct the basic transepithelial ion transport abnormalities and to limit exaggerated inflammatory responses related to the presence of F508del-CFTR protein, they can represent promising compounds for fundamental pharmacotherapy in CF. Since the drugs are in clinical use, therapeutic approaches to address F508del-CFTR defects by PDE5 inhibitors can be considered as a “low-hanging fruit” strategy in the drug discovery tree which could speed up their development as CF therapeutics, as compared to other agents that are under investigation only for CF therapy and for which further exploratory studies are needed before being streamed toward clinical testing. In summary, CFTR correction with PDE5 inhibitors is a promising therapeutic approach based on functional correction of F508del-CFTR activity and on a possible anti-inflammatory action in F508del mice. The effects of these compounds on other CF mutation classes remain to be assessed. The routes for administration should also be further explored, and aerosolized delivery of PDE5 inhibitors should be considered.

## Conflict of Interest Statement

The authors declare that the research was conducted in the absence of any commercial or financial relationships that could be construed as a potential conflict of interest.
